# Central Nervous System and Peripheral Inflammatory Processes in Alzheimer’s Disease: Biomarker Profiling Approach

**DOI:** 10.3389/fneur.2015.00181

**Published:** 2015-08-24

**Authors:** Constance Delaby, Audrey Gabelle, David Blum, Susanna Schraen-Maschke, Amandine Moulinier, Justine Boulanghien, Dany Séverac, Luc Buée, Thierry Rème, Sylvain Lehmann

**Affiliations:** ^1^Laboratoire de Biochimie-Protéomique Clinique, Institute for Regenerative Medicine and Biotherapy (IRMB), CHU de Montpellier and Université Montpellier, Montpellier, France; ^2^Centre Mémoire Ressource Recherche Languedoc Roussillon, Hôpital Gui de Chauliac, CHU de Montpellier, Montpellier, France; ^3^INSERM U837, CHU de Lille, Lille, France; ^4^MGX-Montpellier GenomiX, Institut de Génomique Fonctionnelle, Montpellier, France; ^5^INSERM U1040, CHU de Montpellier, Montpellier, France

**Keywords:** Alzheimer disease, biomarkers, inflammation, cerebrospinal fluid, serum

## Abstract

Brain inflammation is one of the hallmarks of Alzheimer disease (AD) and a current trend is that inflammatory mediators, particularly cytokines and chemokines, may represent valuable biomarkers for early screening and diagnosis of the disease. Various studies have reported differences in serum level of cytokines, chemokines, and growth factors in patients with mild cognitive impairment or AD. However, data were often inconsistent and the exact function of inflammation in neurodegeneration is still a matter of debate. In the present work, we measured the expression of 120 biomarkers (corresponding to cytokines, chemokines, growth factors, and related signaling proteins) in the serum of 49 patients with the following diagnosis distribution: 15 controls, 14 AD, and 20 MCI. In addition, we performed the same analysis in the cerebrospinal fluid (CSF) of 20 of these patients (10 AD and 10 controls). Among the biomarkers tested, none showed significant changes in the serum, but 13 were significantly modified in the CSF of AD patients. Interestingly, all of these biomarkers were implicated in neurogenesis or neural stem cells migration and differentiation. In the second part of the study, 10 of these putative biomarkers (plus 4 additional) were quantified using quantitative multiplex ELISA methods in the CSF and the serum of an enlarged cohort composed of 31 AD and 24 control patients. Our results confirm the potential diagnosis interest of previously published blood biomarkers, and proposes new ones (such as IL-8 and TNFR-I). Further studies will be needed to validate these biomarkers which could be used alone, combined, or in association with the classical amyloid and tau biomarkers.

## Background

Alzheimer disease (AD) is the most common neurodegenerative disorder worldwide. It is characterized by progressive memory loss and cognitive function deficit. Emerging evidences suggest that inflammation plays a central role in AD, and that the pathogenesis of the disease is not restricted to the neuronal compartment, but also involves immunological mechanisms. However, the exact function of inflammation in neurodegeneration is still matter of debate, and it probably has both beneficial and detrimental sides (1).

The chronic inflammatory response occurring in AD patients appears to be triggered by damaged neurons, amyloid beta (Aβ) peptides, and neurofibrillary tangles (2), which are neuropathogenic characteristics of the disease. Inflammation is present from pre-clinical to terminal stages of the disease, as reflected by activated microglia and reactive astrocytes that surround plaques. Microglia activation is a complex phenomenon, resulting in various phenotypes of the cells (secreting different types of cytokines), indicative of their interaction with the environment and allowing for either inflammatory or anti-inflammatory responses. The reactive astrocytes that accumulate around the plaques participate in the clearance of Aβ deposits and in cytokine secretion, thus enhancing the neuro-inflammatory response initiated by microglia [for review, see Ref. (3)].

Diagnosis of AD relies on multidisciplinary approaches, requiring in particular expensive imaging procedures and invasive collection of cerebrospinal fluid (CSF) for biomarkers analysis (4): Aβ (in particular Aβ42), tau, and phospho-tau (p-tau) proteins. Anyway and because of the non-specificity of the symptoms characterizing the disease, its diagnosis is often delayed at a time when the injuries have progressed. The diagnosis of certitude is based on the presence of two neuropathologic processes: neurofibrillary tangles and amyloid senile plaques composed of accumulated tau proteins and Aβ peptides, respectively (5, 6). Despite intensive investigation, there is no cure currently available but only therapies that aim at slowing down the progression of neuronal injuries (4). These drugs are mostly effective at the earliest time course of the disease but are unfortunately administered later, at a time when the diagnosis is defined and injuries have progressed.

Because of the necessity of early diagnosis for optimal treatment and adequate handling of the patients, a reliable signature of specific biomarkers improving identification of pre-clinical AD would be of great interest. If CSF remains the most direct mean to study biochemical changes occurring in the brain, ideal biomarkers should be detectable and measurable in a fluid obtained through less invasive technique, such as the blood. Various groups have recently focused on the search of a plasma panel of AD biomarkers, thus opening promising perspectives in terms of diagnosis of the disease, including at prodromal stages (7–12). However, results were often controversial, because in particular of the heterogeneity of the population-based cohort used and/or the limitation in the sensitivity of the methods used. Thus no blood-based panel has been validated so far as an aid for the diagnosis of AD (13, 14).

Because of the inflammatory component of AD, one can hypothesize that pathological processes associated with this disorder would produce disease-specific molecular changes in the CSF and the blood, as a consequence of the inflammatory mechanisms. Thus, cytokines, chemokines, and growth factors could be expected to be modified, as these are the primary means of communication between cells. They could therefore represent valuable biomarkers for early screening and diagnosis of the disease. To test this hypothesis, we performed multiplex analysis of CSF and serum human samples and simultaneously evaluated the level of expression of 120 biomarkers (corresponding to cytokines, chemokines, growth factors, and related signaling proteins) in these fluids. We discuss our results in this paper, in light of previously published results.

## Materials and Methods

### Sample collection and handling

Blood was collected by venous puncture (BD vacutainer collection tube with clot activator, ref 368815), let it clot at room temperature (RT) for at least 30 min, centrifuged in the next 4 h at 1500 *g* at RT for 10 min. Serum supernatant was subsequently aliquoted by 0.5 mL in 1.5 mL eppendorf microtube (Eppendorf Protein LoBind, ref 0030 108.116) and stored at −80°C until analysis. CSF samples were collected by lumbar puncture in polypropylene tubes (Starstedt; 10 mL, ref 62.610.201), according to standard operating procedures (15), centrifuged at 1000 *g* at 4°C during 10 min, and the supernatant aliquoted and stored as for serum samples.

### Patient description and samples

CSF and sera originated from a sample collection of patients who gave their informed consent from Montpellier neurological and Clinical Research Memory Centers (CMRR) for cognitive or behavioral disorders (officially registered collection DC-2008-417 of the certified NFS 96-900 biobank of the CHRU of Montpellier BB-0033-00031). This study was ethically approved under the number 12.128Ter by the “Comité consultatif sur le traitement de l’information en matière de recherche” (CCTIRS).

Patients were selected based for AD on the clinical criteria established in 1984 by the National Institute of Neurological and Communicative Disorders and Stroke (NINCDS) and the Alzheimer’s Disease and Related Disorders Association (ADRDA) (16). MCI patients were selected following the Petersen MCI diagnosis criteria (17) with a concern regarding a change in cognition, impairment in one or more cognitive domains, preservation of independence in functional abilities without dementia. Mini-mental state examination (MMSE) values illustrating differences in cognition of the different clinical groups are provided in Table 1.

**Table 1:** **Demographic and CSF biomarkers in the population**.

	Diagnosis						*p* (*t*-test)
	Control			AD			
	*N*	Mean	SD	*N*	Mean	SD	
Age	24	66.63	13.30	31	70.84	8.71	0.162
Sex	24	0.67	0.48	31	0.52	0.51	0.17 (Fischer’s test)
IATI	24	1.91	0.72	31	0.95	0.65	<0.001
CSF Aβ42 (pg/mL)	24	1024.58	350.39	31	812.10	314.71	0.059
CSF p-Tau (pg/mL)	24	32.33	10.74	31	87.19	32.74	<0.0001
CSF Tau (pg/mL)	24	188.00	74.27	31	635.97	272.45	<0.0001
CSF protein (g/L)	22	0.49	0.15	29	0.51	0.20	0.397
Serum CRP (mg/L)	21	3.12	4.79	22	2.39	2.64	0.397
Mini-mental state examination (MMSE)/30	20	25.40	6.80	28	22.20	4.80	0.066

*Demographic data, CSF biomarker levels (Aβ42, tau, and p-tau), IATI, CSF proteins, serum CRP (inflammation biomarker), and MMSE are shown for control and AD groups. Results are presented as mean and SD. *t*-test and Fischer exact test were computed*.

For the microarrays approach, a total of 49 serum samples were analyzed. Sera originated from control subjects (*n* = 15), AD (*n* = 14), or MCI (*n* = 20) patients. Among these MCI patients, 10 showed biological characteristics of AD. Of note, the time between the collection of the samples and their analysis was not significantly different between groups (Table 1).We also analyzed the CSF of 20 of these 49 patients (10 AD patients and 10 control subjects).

In the second step, and as a validation study, we selected 31 AD patients (9 of them belonging also to the initial cohort) with a PLM scale of 2 or 3 (18). We also selected 24 control patients (4 of them belonging also to the initial cohort) with a PLM scale of 0 or 1 (18) and the following diagnoses: amyotrophic lateral sclerosis (*n* = 1), Parkinson (*n* = 2), progressive supranuclear palsy (*n* = 2), vascular dementia (*n* = 3), normal pressure hydrocephalus (*n* = 2), Lewy body dementia (*n* = 2), peripheral neuropathy (*n* = 1), and subjective cognitive impairment (*n* = 11).

### Protein-arrays analysis

The relative abundance of 120 known signaling proteins (Table S1 in Supplementary Material) was measured in the 69 biological samples (49 sera and 20 CSF) using protein antibodies-based arrays (RayBio^®^ Human Cytokine Antibody Array G-Series 1000, AAH-CYT-G1000-8). Antibodies used for detection of the 120 proteins are distributed on two slides (G6 and G7), each one allowing the semi-quantitation of 60 of the signaling proteins (see Table S1 in Supplementary Material for protein maps). Every sample tested was thereafter and simultaneously hybridized on two slides: G6 and G7.

One hundred microliters of native CSF or diluted serum (1:2.5) of each patient was hybridized on the slides, according to the provider’s recommendations. As an internal quality control, a pool of five sera (originating from control patients) was prepared in our laboratory and hybridized on every slide, thus ensuring the control of the homogeneity of the results between the arrays. Slides were scanned at 532 nm (GenePix 4200AL, Axon instruments).

All the numeric data obtained following scan of the arrays were normalized according to the manufacturer’s recommendations [using the normalization file provided with the kit (AAH-CYT-G1000-8, RayBio^®^)].

### Quantitative analysis through ELISA and electrochemiluminescence assays

Quantitative multiplex or simplex methods were performed in both the CSF and serum of 55 patients (31 AD patients and 24 control subjects), using either electrochemiluminescence (MesoScaleDiscovery technology, MSD, Sector Imager 2400A) or ELISA method. Quantification of FABP3, TIMP-1, MIP-1beta, and RANTES was performed using simplex detection MSD kit, while GRO-alpha, IL-8, MCP-1, MIP-3beta, and sTNFR-I were simultaneously measured using MSD custom V-Plex detection (MSD MULTI-SPOT^®^ 7 Spot Special Order Human 5Plex). Quantification of IGFBP-6, sIL-6R, IL-3, and MIP-1alpha was performed through simplex ELISA, purchased from Clinisciences. CSF samples were measured directly, without previous dilution. Depending on the cytokine measured, serum samples were diluted (1:2–1:50).

### Statistical analysis

For protein-arrays analysis, prediction analysis for microarrays (PAM) was performed with normalized array measurements of the 120 signaling proteins quantified in the training set (software R 3.1). To minimize the risk of overfitting, the PAM approach used as a training set 90% of the population, and as a validation the remaining 10%. This cross-validation was repeated 10 times. For exploitation of quantitative ELISA results, Student’s *t*-test and area under ROC curves (AUC) analysis were performed using medCalc^®^ software ver 15.2.2. The logistic regression was achieved with the same software with backward stepwise selection using a significance level of 0.10. Classification trees were obtained using a Microsoft Visual Studio routine (available upon request), which computed the sensitivity and specificity of all possible pair combination of biomarkers at the different cut-offs (corresponding to the values of the biomarkers in the population).

## Results

### Semi-quantitative analysis of 120 proteins through protein-arrays approach

**Figure 1:**
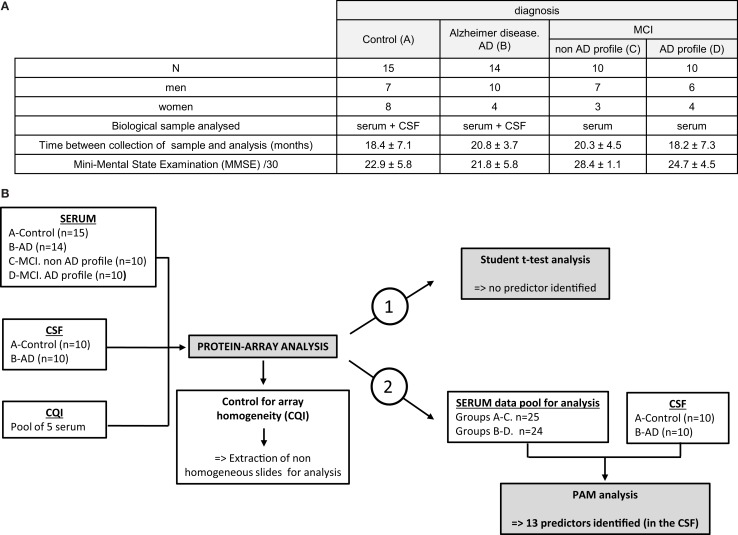
**Cohort patients and study outline**. **(A)** A total of 49 patients were included in the preliminary study for protein-arrays analysis: 15 control subjects (A), 14 AD late-stage patients (B) and 20 MCI (mild cognitive impairment): 10 MCI with non-AD profile (C) and 10 MCI presenting AD profile (D). Serum was analyzed for every patient included; CSF was analyzed for 20 of them (10 patients from groups A and B). Time between collection of samples (serum and CSF) and their analysis is indicated (in months): results are mean ± SD. Mini-mental state examination (MMSE) is indicated (MMSE/30) as mean ± SD. **(B)** Sera, CSF, and our internal quality control (CQI, corresponding to a pool of five control sera) were hybridized on the protein arrays. Homogeneity of the slides was controlled thanks to the CQI: non-homogeneous slide identified was extracted before proceeding to the analysis of the arrays. Normalized array data of 120 serum signaling proteins were analyzed in the training set with statistical Student’s *t*-test to discover differences in protein abundance between samples (strategy 1). As no predictor could be identified, serum data were pooled for groups A–C and B–D (strategy 2). To discover predictors for classification, the training set was analyzed through prediction analysis for microarrays (PAM) approach.

Forty-nine patients of clinically characterized diagnosis were included for the protein-array analysis: the cohort was composed of individuals with pre-symptomatic (MCI, mild cognitive impairment, *n* = 20) or late-stage AD (*n* = 14) patients and from control subjects (*n* = 15), Figure 1A. MMSE differed between the groups and was, as expected, significantly correlated with Aβ42, Tau or p-Tau (*p* < 0.001, “Spearman” rank correlation).

The serum of these 49 patients and the CSF of 20 of them (10 controls and 10 AD) were hybridized simultaneously on G6 and G7 slides, in order to evaluate the relative abundance of the 120 proteins detectable on the arrays (Figures 1A,B, left panel). Before proceeding to the analysis of the slides and to ensure for their homogeneity, an internal quality control (CQI) was hybridized on every array, in the very same conditions than biological samples included in that study. This CQI corresponds to a pool of sera (originated from control subjects) prepared in our laboratory and was used to compare and homogenize the slides, so that the fluorescence detected could be attributed to specific variation of expression of the proteins in a sample, rather than a slide effect (Figure 1B, left and central panels and Data S1 in Supplementary Material). Our data show that CQI inter-slide are homogenous between G7 slides, which validated the subsequent analysis of numeric data obtained for the proteins studied on these arrays (Data S1 in Supplementary Material, left graph). On the other hand, analysis of CQI on G6 slides showed that one of these arrays gave non-homogenous results (Data S1, right graph, gray-highlighted results); the corresponding slide was thereafter extracted before proceeding to the subsequent analysis (Figure 1B, central panel).

Following this preliminary control of the slides, normalized data generated from the 49 sera and 20 CSF hybridized were analyzed through semi-quantitative protein-arrays approach: the relative abundance of 120 proteins of known function (cytokines, chemokines, and other signaling proteins) was simultaneously evaluated (Figure 1B, right panel).

**Figure 2:**
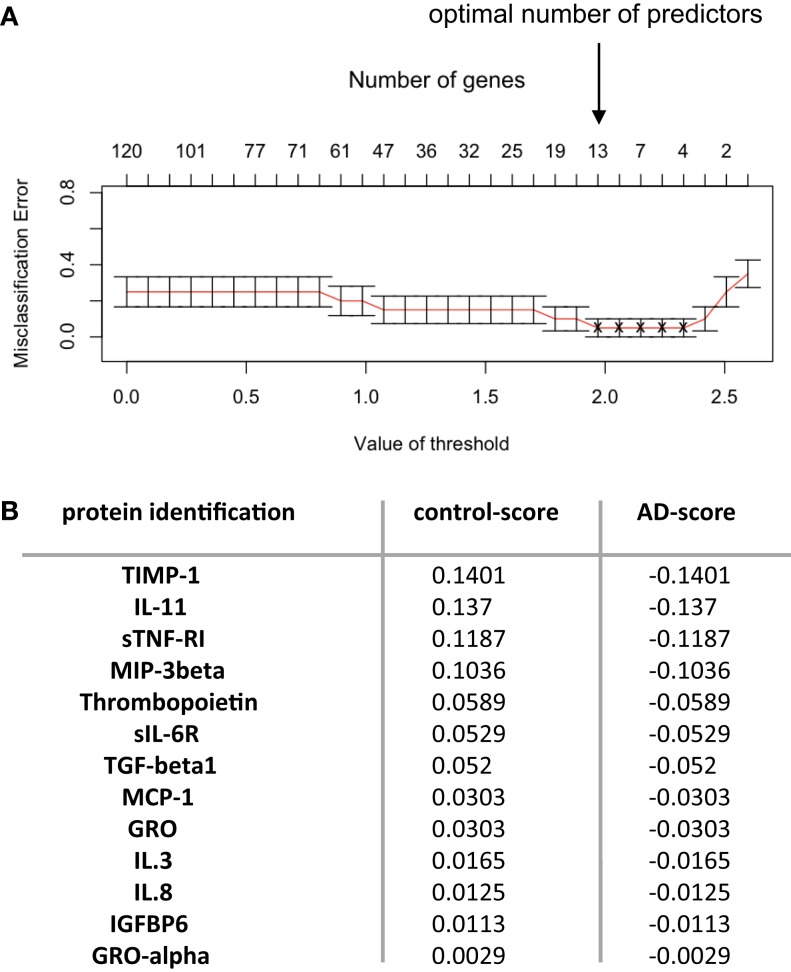
**Predictors discovery**. **(A)** Predictor discovery by prediction analysis for microarrays (PAM) was performed with normalized array measurements of 120 signaling proteins in the training set. Internal cross-validation (redline) decreasing the centroid threshold (lower *x*-axis) resulted in an increase in the number of markers (inserted upper *x*-axis) that were used for classification and calculation of the classification error (*y*-axis). This led to the discovery of an optimal set of 13 predictors with lowest possible classification error. **(B)** The 13 predictors identified through PAM analysis are presented. Proteins are arranged in columns, with *d*-score corresponding in each group. Control group corresponded to control subjects and MCI non-AD patients (groups A–C defined for protein-arrays analysis); AD group corresponded to AD late-stage patients and MCI presenting AD profile (groups B–D defined for protein-arrays analysis). Positive *d*-score is indicative of increased expression and negative *d*-score reflects decrease in the expression of the proteins analyzed.

The numeric and normalized data obtained from the protein arrays were first of all analyzed through Student’s *t*-test, in order to identify a set of putative biomarkers in the serum that could participate to the discrimination of control, MCI, and late-stage AD patients (Figure 1B, right and up panel); however, this statistical analysis did not provide exploitable results, as no biomarker appeared to be significantly and specifically associated to one group more than another one (results not shown). Furthermore, we decided to pool together the data obtained with the serum of all the AD patients (MCI with AD profile and late-stage) and control patients (control and MCI no AD profile) and to focus on the discrimination between control and AD patients (Figure 1B, right panel strategy 2). Prediction analysis for microarrays (PAM) was thereafter performed with the normalized array measurements. In the serum, we were still unable to identify proteins significantly modified between the two groups. On the other hand, the same analysis in the CSF led to the discovery of a repeatedly optimal set of 13 predictors giving the lowest possible classification error between control and AD groups (Figures 2A,B). Results showed that all of them were decreased in AD group (negative *d*-score, Figure 2B).

### Quantitative analysis of putative biomarkers in the CSF and the serum

**Figure 3:**
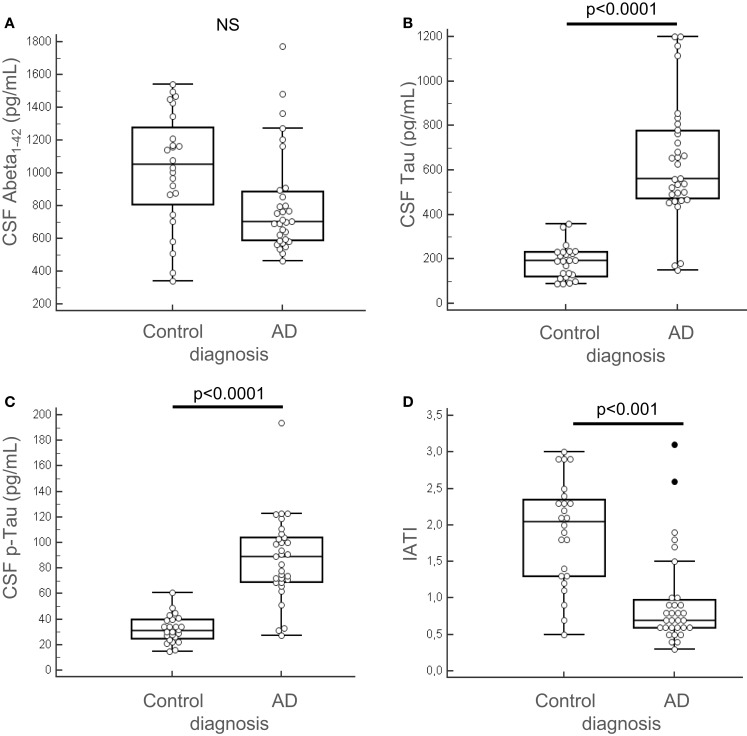
**Clinical biochemical characterization of patients**. The 55 patients (AD, *n* = 31 and control subjects, *n* = 24) of the enlarged cohort used for the second part of the study were characterized clinically and quantified for CSF biomarkers: Aβ42, tau, and p-tau, using Fujirebio ELISA quantification kits **(A–C)**. IATI ratio [Aβ42/(240 + 1,18tau)] **(D)** was calculated for every subject of the cohort. Outliers are indicated with black circle. Outliers are defined as a value that is smaller than the lower quartile minus three times the interquartile range, or larger than the upper quartile plus three times the interquartile range.

Among the 13 putative proteins of interest identified in the CSF, 10 of them (plus 4 additional selected following literature) were subsequently analyzed through quantitative method in the CSF and the serum of AD and control patients. This quantification was performed on an enlarged cohort, composed of 55 individuals (31 AD patients and 24 control subjects), characterized clinically and biologically for CSF biomarkers: Aβ42, tau, and p-tau (Table 1; Figure 3). According to their clinical diagnosis, AD patients showed a decrease of CSF Aβ_1–42_, together with an increase of CSF tau and p-tau (*p* < 0.0001). The ratio IATI [Aβ_1–42_/(240 + 1.18 × tau)] was calculated for these patients and allowed for diagnostic discrimination of the two groups (*p* < 0.001, Figure 3D). Groups were homogeneous in terms of age, sex repartition, CRP, and CSF protein, and MMSE was decreased in AD group (Table 1).

**Figure 4:**
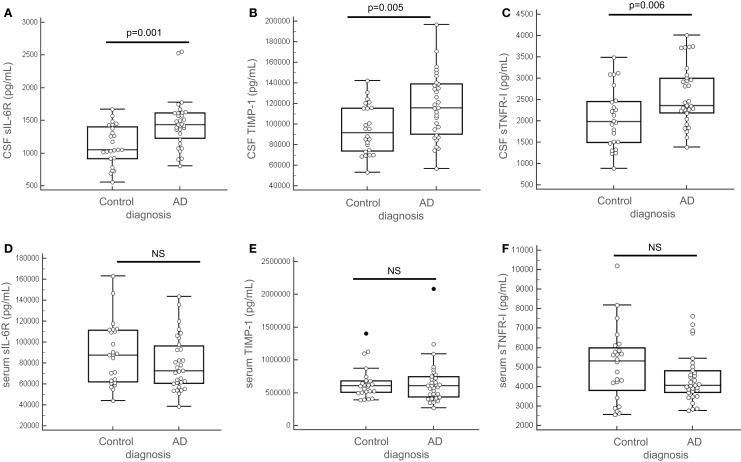
**Predictors quantification in the CSF and the serum**. sIL-6R, TIMP-1, and sTNFR-I were quantified in the CSF **(A–C)** and in the serum **(D–F)** of 55 patients (AD, *n* = 31 and control subjects, *n* = 24). Outliers are indicated with black circle. Outliers are defined as a value that is smaller than the lower quartile minus three times the interquartile range, or larger than the upper quartile plus three times the interquartile range.

We tested for normal distribution of the data concerning the 14 proteins measured and thereafter proceeded for *t*-student statistical test to evaluate significant difference between AD and control groups (Table 2). Results showed that in the CSF, among the 14 tested proteins, 3 biomarkers were significantly different between the two groups (Table 2, bolded and gray-highlighted results): sIL-6R, TIMP-1, and sTNFR-I. These three proteins were increased in the CSF of AD patients, compared to control subjects (Figures 4A–C). Furthermore, three of the 14 biomarkers quantified were undetectable in the CSF of patients (FABP3, GRO-gamma, and TGF-beta1). On the other side, all of the 14 biomarkers tested were detectable in the serum but none of them were detected at a level significantly different between AD and control patients (Table 2; Figures 4D–F).

**Table 2:** **CSF and serum quantification of predictors identified**.

		Diagnosis						*p* (*t*-test)
		Control			AD			
		*N*	Mean	SD	*N*	Mean	SD	
CSF	FABP-3 (pg/mL)	24	ND	ND	31	ND	ND	–
	GRO-alpha (pg/mL)	24	22.79	12.28	31	29.97	18.63	0.101
	GRO-gamma (pg/mL)	24	ND	ND	27	ND	ND	–
	IGFBP6 (pg/mL)	24	76,4473.50	680,240.30	31	65,6967.45	317,572.10	0.440
	IL-3 (pg/mL)	24	71.08	55.42	31	81.94	141.47	0.721
	IL-8 (pg/mL)	24	2476.46	2164.15	31	3398.90	3307.04	0.242
	MCP-1 (pg/mL)	24	484.54	110.74	31	524.87	221.39	0.420
	MIP-1beta (pg/mL)	24	11.38	7.89	31	11.58	3.09	0.957
	MIP-3beta (pg/mL)	24	193.33	137.71	31	249.97	110.29	0.096
	RANTES (pg/mL)	24	4.25	8.36	31	9.81	20.49	0.210
	sIL6-R (pg/mL)	**24**	**1125.71**	**304.31**	**31**	**1455.42**	**384.44**	**0.001**
	TGF-beta1 (pg/mL)	24	ND	ND	31	ND	ND	–
	TIMP-1 (pg/mL)	**24**	**94,953.75**	**23,724.34**	**31**	**117,507.10**	**31,685.66**	**0.005**
	sTNFR-I (pg/mL)	**24**	**2058.88**	**700.90**	**31**	**2601.94**	**693.10**	**0.006**
Serum	FABP-3 (pg/mL)	24	7.13	2.47	31	6.36	2.44	0.247
	GRO-alpha (pg/mL)	23	106.50	64.10	31	111.10	56.08	0.777
	GRO-gamma (pg/mL)	24	98.91	231.92	27	128.44	258.91	0.676
	IGFBP6 (pg/mL)	24	887,849.71	565,626.11	31	717,297.23	325,456.73	0.306
	IL-3 (pg/mL)	24	2353.25	7191.47	31	1242.19	1409.37	0.372
	IL-8 (pg/mL)	24	21.42	17.54	31	17.36	16.77	0.392
	MCP-1 (pg/mL)	24	319.21	100.39	31	319.13	99.70	0.993
	MIP-1beta (pg/mL)	24	28.67	13.78	31	28.45	16.95	0.957
	MIP-3beta (pg/mL)	24	230.71	452.88	31	154.94	188.86	0.403
	RANTES (pg/mL)	24	189.58	93.68	31	216.16	117.38	0.368
	sIL6-R (pg/mL)	24	88,788.79	30,096.89	31	79,709.42	26,040.66	0.236
	TGF-beta1 (pg/mL)	24	1.13	0.34	31	1.26	0.44	0.677
	TIMP-1 (pg/mL)	24	65,6950	248,683.10	31	660,009.68	342,382.44	0.971
	sTNFR-I (pg/mL)	24	5139.29	1880.56	31	4424.97	1242.77	0.096

*Ten predictors previously identified following PAM analysis and four supplemental ones (FABP3, GRO gamma, RANTES, and MIP-1beta) were quantified in the CSF and the serum of AD (*n* = 31) and control (*n* = 24) patients using quantitative ELISA and MSD approaches. Results are presented as mean and SD. The three bolded and gray-highlighted biomarkers present significant difference in the CSF between control and AD groups (Student’s *t*-test). None of the proteins tested in the serum show significant difference between the two groups. ND corresponds to proteins undetectable in the CSF*.

### ROC curves, regression analysis and decision trees of the biomarkers in the serum and the CSF

**Figure 5:**
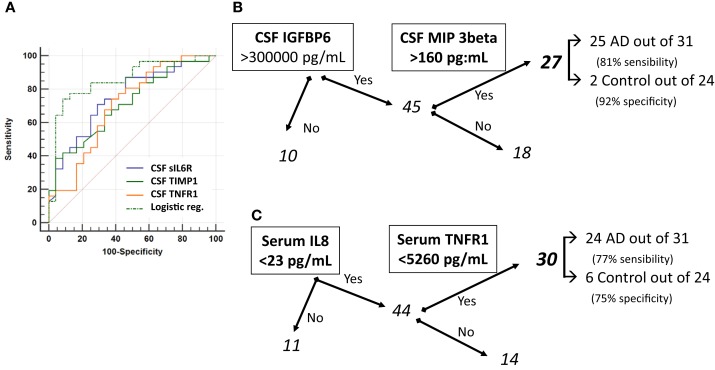
**Area under ROC curve (AUC) of Tau, p-Tau, and sTNFR-I**. ROC curves **(A)** of the three most efficient biomarkers for AD diagnosis sIL-6R, TIMP-1, and sTNFR-I were plotted along with their combination (logistic regression, see text). The classification tree for CSF biomarkers **(B)** defined as AD samples those with IGFBP6 >300,000 pg/mL and MIP-3beta >160 pg/mL. Using serum biomarkers **(C)**, the criteria selected for AD diagnosis was: IL8 <23 pg/mL and TNFR-I <5260 pg/mL.

We compared the area under the ROC curves (AUC) of all the analytes measured in the serum and the CSF of the 55 patients (Table 3). CSF tau and p-tau appeared to be the most efficient analytes to discriminate AD patients and control subjects (AUC values = 0.942 and 0.946, respectively). Among the analytes tested, seven presented an AUC ≥ 0.655 (Table 3, bolded and highlighted results). To combine these CSF biomarkers, we tested a logistic regression model which retained three biomarkers with the following equation: sIL6R × 0.0034615 + TIMP1 × 0.000024458 + TNFRI × 0.001016 − 9.2101 (pg/mL). As illustrated in Figure 5A, this resulted in an important improvement of the AUC reaching 0.858 (corresponding at its best to a sensitivity of 74.2% and a specificity of 91.7%). The relevance of these biomarkers for AD was also supported by the fact that a significant “Spearman” rank correlation was observed between MMSE and CSF TIMP-1 (*p* = 0.03950) and between tau or p-tau and CSF sIL6R (*p* < 0.001). The low differences in expression of the biomarkers in the blood prevented their integration in the logistic regression model.

**Table 3:** **Values of area under the ROC curves**.

	Variable	AUC	SE	95% CI
CSF	IATI	0.788	0.0739	0.636–0.898
	Abeta 1–42	0.69	0.0789	0.550–0.807
	GRO-alpha	0.604	0.0794	0.463–0.733
	IGFBP6	0.511	0.0867	0.372–0.648
	IL-3	0.566	0.0748	0.425–0.699
	IL-8	0.614	0.0792	0.473–0.742
	MCP-1	0.503	0.0791	0.365–0.641
	**MIP-1beta**	**0.655**	**0.0776**	**0.514–0.778**
	**MIP-3beta**	**0.727**	**0.0787**	**0.590–0.839**
	**p-Tau**	**0.946**	**0.0315**	**0.849–0.989**
	RANTES	0.618	0.0748	0.477–0.745
	**sIL6-R**	**0.755**	**0.0659**	**0.620–0.861**
	**Tau**	**0.942**	**0.034**	**0.843–0.987**
	**TIMP-1**	**0.692**	**0.0756**	**0.544–0.816**
	**sTNFR-I**	**0.699**	**0.0771**	**0.551–0.821**
	CSF protein	0.514	0.0854	0.367–0.660
Serum	CRP	0.512	0.0924	0.355–0.667
	Fabp-3	0.646	0.0817	0.497–0.778
	GRO-alpha	0.57	0.0873	0.421–0.710
	GRO-gamma	0.579	0.076	0.430–0.719
	IGFBP6	0.586	0.0801	0.445–0.717
	IL-3	0.549	0.0797	0.409–0.684
	IL-8	0.599	0.081	0.459–0.729
	MCP-1	0.501	0.0806	0.363–0.639
	MIP-1beta	0.548	0.0821	0.408–0.683
	MIP-3beta	0.622	0.0761	0.481–0.750
	RANTES	0.556	0.079	0.416–0.690
	sIL6-R	0.595	0.0789	0.455–0.726
	TGF-beta1	0.567	0.0528	0.426–0.700
	TIMP-1	0.52	0.0795	0.381–0.657
	sTNFR-I	0.625	0.0838	0.484–0.752

*Area under the ROC curve (AUC) for all the biochemical analytes quantified in the 55 patients (AD, *n* = 31 and control subjects, *n* = 24) of the study was evaluated. The seven biomarkers bolded and gray-highlighted analytes present AUC ≥ 0.650*.

We also tested a simple classification tree model based on only two analytes (nodes) to minimize the risk of overfitting (Figures 5B,C). For the CSF biomarkers, this resulted in a classification involving IGFBP6 and MIP-3 beta, reaching on their own a sensitivity of 81% and a specificity of 92% (Figure 5B). Interestingly, the logistic regression and the classification tree resulted in a different biomarker selection and the patients selected with the two models were also different. Additional studies on larger cohort will be needed to reconcile and eventually combine these results. Applying the same approach for serum biomarkers, it resulted in a classification involving IL-8 and TNFR-1, reaching a sensitivity of 77% and a specificity of 75% (Figure 5C).

Of note, the control misclassified patients of the classification trees (Figures 5B,C) corresponded to various diagnoses (Lewy body dementia, vascular dementia, and subjective cognitive impairment). Based on the available clinical and biological data, it was not apparent why these patients were misclassified.

## Discussion

The 2011 revision of criteria for AD clinical diagnosis includes CSF biomarkers analysis: quantification of Aβ42 peptides, tau, and p-tau proteins; however and despite its utility, its use in routine clinical practice and for the follow-up of patient is limited because in particular of the invasive character of lumbar puncture.

The link between neuro-inflammation and AD has opened attractive perspectives for the early diagnosis and handling of patients. Indeed, the possibility to identify a blood-based panel of biomarkers to detect AD patients could allow for a systematic and early diagnosis of them, at the time of the first signs indicative of cognitive impairment, therefore optimizing their care and treatment. In addition, the perspective of feasibility of an early biochemical diagnosis of patients through minimally invasive technique (such as venous puncture) is quite seducing.

Various studies described such a signature in the blood: among them, Ray et al. identified 18 blood biomarkers, some of which were subsequently confirmed by ADNI (8, 19). The present work aimed at investigating the molecular mechanisms involved in inflammatory processes occurring in AD, intending to identify or confirm a profile of biomarkers characteristic of AD. To this end, the modification of various signaling proteins (chemokines and cytokines) in the CSF and the serum of AD patients were evaluated using multiplex strategies.

The protein-arrays approach used in the first place is very attractive because it offers the opportunity to evaluate, in a single test and through a reduced volume of biological sample, the simultaneous level of expression of numerous proteins. In the present study, the large screening of 120 signaling proteins in serum and CSF of AD patients seemed very promising but appeared unfortunately quite disappointing in the serum. Indeed, no putative biomarker could be identified through this approach. One cannot exclude the possibility that the protein-arrays approach might lack sensitivity and reproducibility to detect small and discrete differences in the level of expression of the proteins tested between the groups of our cohort. Anyway such observation remains quite intriguing because other studies, using similar approaches, described a blood-based panel of biomarkers discriminating control subjects and AD patients (8). However, a strict comparison of these works remains challenging because of the heterogeneity and the different size of the cohorts used, and also because of the nature of the samples used (serum versus plasma). On the other hand, the technique provided interesting results in the CSF, as 13 putative biomarkers potentially discriminating control subjects and AD patients could be identified. Although such method is very useful as a first-step and large screening of candidates, it remains semi-quantitative and poorly sensitive. Thus, the putative biomarkers identified in the CSF had to be subsequently tested for confirmation through quantitative and sensitive methods.

Among the 13 predictors identified, 10 were analyzed through such quantitative approaches, according to the availability of the existent kits: GRO-alpha, IGFBP6, IL-3, IL-8, MCP-1, MIP-3beta, sIL-6R, TGF-beta1, TIMP-1, and sTNF-RI. Because of their potential implication in inflammatory processes and AD, we also evaluated the abundance of four supplemental proteins present in our panels: GRO-gamma, RANTES, MIP-1beta, and FABP3, which were also described in the literature as putative AD biomarkers. These 14 proteins were quantified in both the CSF and the serum of an enlarged cohort of 55 subjects (AD patients and control individuals). Among these biomarkers, three presented significant increase in the CSF: sIL-6R, TIMP-1, and sTNFR-I, and could be combined in a logistic regression model. Interestingly, sIL-6R and sTNF-RI presented opposite way of variation in the serum, although not being significant in this fluid.

Microglia and astrocytes are the major sources of cytokines production in AD. Thus, Aβ42 accumulation has been suggested to be a strong inducer of the neuro-inflammatory response in AD, exposure of microglia to Aβ42 deposits increasing production of IL-6 and M-CSF (20). M-CSF was also described to be increased in the plasma and the CNS of patients at the dementia stage of AD compared to control or MCI age-matched patients (20, 21). Controversial results were obtained by Ray et al., describing a decrease of M-CSF in the plasma of AD patients (8). In our study, this cytokine presented a very low basal level and did not show any significant modification of level neither in the CSF or serum of our patients (protein-array results). In addition, IL-6 was barely detectable in the CSF and serum of our patients but as noticed above, its receptor (sIL-6R) was significantly increased in the CSF and showed a tendency to decrease in the serum of AD patients although not being significant. A CSF cytokine profile characterized by an increase of TNF-alpha associated with a decrease of TGF-beta could be a marker of the conversion of MCI to AD (22). In addition, TNF-alpha was reported to be decreased in the plasma of AD patients versus control (8). In our study anyway, we did not observe significant change in the CSF or the serum of TNF-alpha and TGF-beta level among the patients. Interestingly, the receptor of TNF-alpha (sTNFR-I) was significantly increased in the CSF of AD patients compared to control subjects and showed a tendency to decrease in the serum of AD patients. Of note, He et al. recently demonstrated that deletion of TNF-RI can inhibit Aβ generation and prevents cognitive deficits in AD mice (23), through the reduction of expression and activity of BACE1 mediated by NF-κB signaling. Thus, chronic overexpression of neuronal TNF-alpha has been described to enhance local inflammatory responses in transgenic AD mice (24). However, the pro-inflammatory cytokine TNF-alpha is also reported to present neuroprotective effects in the brain (25). In addition and very interestingly, analysis of AUC of ROC curves in our study shows that association of sTNF-RI was among the best biomarkers and that its combination with TIMP-1 and sILR-6 provides the most powerful combination for AD diagnosis. Such observation will have to be confirmed in another study through an enlarged cohort.

On the other side, IL-3 is described in the literature to be reduced in the plasma of AD patients, which was also observed in our cohort (although not being significant). In addition, IL-1beta is known to be secreted by activated microglia cells following Aβ42 stimulation *in vitro* (26). Thus this pro-inflammatory cytokine can be detected in microglial cells surrounding Aβ deposits and in the CSF of AD patients (26). Anyway, only very low level of this cytokine was detectable in the CSF and serum of our patients and no difference in its concentration could be noted among the patients. IGFBP6 is also described to be increased in the plasma of AD patients (8) but we did not detect significant changes of its level in our study. Interestingly, this molecule was retained in the classification tree model, which would need further validation in a larger cohort.

Chemio-attractive chemokines are known to participate in the inflammatory process of AD, through regulation of microglial cells migration at the site of inflammation (27). In particular, CCL4 (MIP-1beta) has been described in reactive astrocytes surrounding Aβ deposits (28) and CXCL8 (IL-8), CCL2 (MCP-1), and CCL3 (MIP-1alpha) are increased following Aβ42 exposition of astrocytes (29, 30). IL-8 has also previously been described to be increased in the plasma of AD patient (8). In our study, we detected increase of IL-8 and MCP-1 in the CSF of AD patients and no significant modification of MIP-1alpha or beta, neither in the CSF nor the serum.

On the other side, CCL5 (RANTES) was described to be down-regulated in the plasma of AD patients (8) and we noted no significant variation of this chemokine between the two groups of our study. GRO-alpha has also been reported to be a CSF biomarker of interest for AD diagnosis (31) but its level remained unchanged between our two groups. GRO-gamma (CXCL3) was undetectable in the CSF of our patients and remained unchanged in the serum among the patients.

In addition, matrix metalloproteinases (MMP) are believed to be involved in the pathologic processes of AD. TIMP-1 is the tissue inhibitor of MMP-9 and has been described to be increased in the CSF of AD and MCI patients (32). In our study, TIMP-1 was also significantly increased in the CSF of AD patients. Its level appeared stable in the serum of all populations of our cohort.

Finally, obesity, defined as a disorder in which excess fat accumulates in the body, also induces chronic inflammatory processes. Indeed, obesity has been associated with higher risk to develop AD (33). FABP3 is a member of the fatty acid binding proteins and has recently been described to be down-regulated in the brain of AD patients (34). However, FABP3 was not detectable in the CSF and presented no significant variation in the serum of our patients.

Identification of a molecular signature for AD diagnosis is very promising in terms of handling of patients and early diagnosis of AD, but remains quite challenging. Indeed, numerous studies have described panels of biomarkers of potential interest but at the time of their confirmation, results appeared to be largely controversial [for review, see Ref. (3)]. Our study led us to identify three differential biomarkers in the CSF of AD patients (sIL-6R, TIMP-1, and sTNFR-I), which could be efficiently combined. They were, however, not differential in the serum. On the other hand, using classification trees, we could obtain notable results in both CSF and serum, involving, respectively, IGFBP6 and MIP-3 beta or IL-8, and TNFR-I. Upon confirmation, these results could represent interesting new means for the diagnostic of AD. These biomarkers, associated with the biochemical diagnostic tools currently used for AD diagnosis (such as CSF biomarkers Aβ, tau, and p-tau), could be of particular interest for early diagnosis of AD or for patients presenting ambiguous profiles.

In conclusion, this study confirms the potential diagnosis interest of previously published blood biomarkers, and proposes new ones (such as IL-8 and TNFR-I). Further studies will be needed to validate these biomarkers which could be used alone, combined, or in association with the classical CSF biomarkers.

## Conflict of Interest Statement

The authors declare that the research was conducted in the absence of any commercial or financial relationships that could be construed as a potential conflict of interest.

## Supplementary Material

The Supplementary Material for this article can be found online at http://journal.frontiersin.org/article/10.3389/fneur.2015.00181

Table S1 **Antibody array map of G6 and G7 slides**. Each array allowed for the detection and semi-quantitation of 60 human cytokines. POS and NEG correspond to positive and negative control, respectively, and are used for normalization of fluorescence detected from the slides.

Data S1 **CQI homogeneity between arrays**. CQI was hybridized on each array (G6 and G7) in the very same conditions than the biological samples studied. Non-homogenous slide (G6, gray-highlighted) was extracted before analysis of the normalized data generated.
