# Supplementary material for: Central Nervous System and Peripheral Inflammatory Processes in Alzheimer’s Disease: Biomarker Profiling Approach
Source: Front Neurol. 2015 Aug 24;6:181. doi: 10.3389/fneur.2015.00181 (PMC4547499; doi:10.3389/fneur.2015.00181)
Supplement: Table S1 — Antibody array map of G6 and G7 slides. Each array allowed for the detection and semi-quantitation of 60 human cytokines. POS and NEG correspond to positive and negative control, respectively, and are used for normalization of fluorescence detected from the slides. [file Table_1.PDF]

Supplementary table 1.

G6 array map
(60 human cytokines detected)

ANG = Angiogenin, OSM = Oncostatin M, THPO = Thrombopoietin, CCL11 = Eotaxin, CCL24 = Eotaxin-2, CCL26 = Eotaxin-3, Flt-3L = Flt-3 Ligand, CX3CL1 = Fractalkine, CSF2 = GM-CSF, CSF1 = M-CSF
TGF-β1 reacts only with active form of TGF-β1

	A	B	C	D	E	F	G	H	I	J	K	L	M	N
1	POS1	POS2	POS3	NEG	NEG	ANG	BDNF	BLC	BMP-4	BMP-6	Ckβ 8-1	CNTF	EGF	CCL11
2	POS1	POS2	POS3	NEG	NEG	ANG	BDNF	BLC	BMP-4	BMP-6	Ckβ 8-1	CNTF	EGF	CCL11
3	CCL24	CCL26	FGF6	FGF7	Flt-3L	CX3CL1	GCP-2	GDNF	CSF2	I-309	IFN-γ	IGFBP1	IGFBP2	IGFBP4
4	CCL24	CCL26	FGF6	FGF7	Flt-3L	CX3CL1	GCP-2	GDNF	CSF2	I-309	IFN-γ	IGFBP1	IGFBP2	IGFBP4
5	IGF-I	IL-10	IL-13	IL-15	IL-16	IL-1α	IL-1β	IL-1ra	IL-2	IL-3	IL-4	IL-5	IL-6	IL-7
6	IGF-I	IL-10	IL-13	IL-15	IL-16	IL-1α	IL-1β	IL-1ra	IL-2	IL-3	IL-4	IL-5	IL-6	IL-7
7	Leptin	LIGHT	MCP1	MCP2	MCP3	CSF1	MDC	MIG	MIP-1δ	MIP-3α	NAP-2	NT-3	NT-4	PARC
8	Leptin	LIGHT	MCP1	MCP2	MCP3	CSF1	MDC	MIG	MIP-1δ	MIP-3α	NAP-2	NT-3	NT-4	PARC
9	PDGF-BB	RANTES	SCF	SDF-1	TARC	TGF-β1	TGF-β3	TNF-α	TNF-β	NEG	NEG	NEG	NEG	NEG
10	PDGF-BB	RANTES	SCF	SDF-1	TARC	TGF-β1	TGF-β3	TNF-α	TNF-β	NEG	NEG	NEG	NEG	NEG

G7 array map
(60 human cytokines detected)

Acrp30 = Adiponectin, AgRP = Agouti-related Protein, ANGPT2 = Angiopoietin-2, AREG = Amphiregulin, bFGF = basic FGF (FGF2), BTC = Betacellulin, CCL28 = MEC, CSF3 = G-CSF, GITRL = GITR Ligand, XCL1 = Lymphotactin, OPG = Osteoprotegerin, OSM = Oncostatin M, PLGF = Placental Growth Factor, THPO = Thrombopoietin
GRO recognizes CXLC1, CXCL2 and CXCL3 (GRO-α, GRO-β and GRO-γ, respectively)
sIGF-1R, sIL-6R, sTNFR1, sTNFR2, sgp120, TRAIL R3 and TRAIL R4 recognize soluble receptors.
VEGF-A detects VEGF(165 aa) and VEGF(121 aa).

	A	B	C	D	E	F	G	H	I	J	K	L	M	N
1	POS1	POS2	POS3	NEG	NEG	Acrp30	AgRP	ANGPT2	AREG	Axl	bFGF	β-NGF	BTC	CCL28
2	POS1	POS2	POS3	NEG	NEG	Acrp30	AgRP	ANGPT2	AREG	Axl	bFGF	β-NGF	BTC	CCL28
3	CTACK	Dtk	EGFR	ENA-78	Fas	FGF4	FGF9	CSF3	GITRL	GITR	GRO	GRO-α	HCC-4	HGF
4	CTACK	Dtk	EGFR	ENA-78	Fas	FGF4	FGF9	CSF3	GITRL	GITR	GRO	GRO-α	HCC-4	HGF
5	ICAM1	ICAM3	IGFBP3	IGFBP6	sIGF-1R	IL1R4	IL-1 R1	IL-11	IL-12 p40	IL-12 p70	IL-17	IL-2 Rα	sIL-6R	IL-8
6	ICAM1	ICAM3	IGFBP3	IGFBP6	sIGF-1R	IL1R4	IL-1 R1	IL-11	IL-12 p40	IL-12 p70	IL-17	IL-2 Rα	sIL-6R	IL-8
7	I-TAC	XCL1	MIF	MIP-1α	MIP-1β	MIP-3α	MSP-α	NT-4	OPG	OSM	PLGF	sgp130	sTNFR2	sTNFR1
8	I-TAC	XCL1	MIF	MIP-1α	MIP-1β	MIP-3α	MSP-α	NT-4	OPG	OSM	PLGF	sgp130	sTNFR2	sTNFR1
9	TECK	TIMP1	TIMP2	THPO	TRAIL R3	TRAIL R4	uPAR	VEGF-A	VEGF-D	NEG	NEG	NEG	NEG	NEG
10	TECK	TIMP1	TIMP2	THPO	TRAIL R3	TRAIL R4	uPAR	VEGF-A	VEGF-D	NEG	NEG	NEG	NEG	NEG
