# Supplementary figures and images for: Central Nervous System and Peripheral Inflammatory Processes in Alzheimer’s Disease: Biomarker Profiling Approach
Source: Front Neurol. 2015 Aug 24;6:181. doi: 10.3389/fneur.2015.00181 (PMC4547499; doi:10.3389/fneur.2015.00181)

## Supplementary data 1.

Homogeneity of CQI inter G7-slides

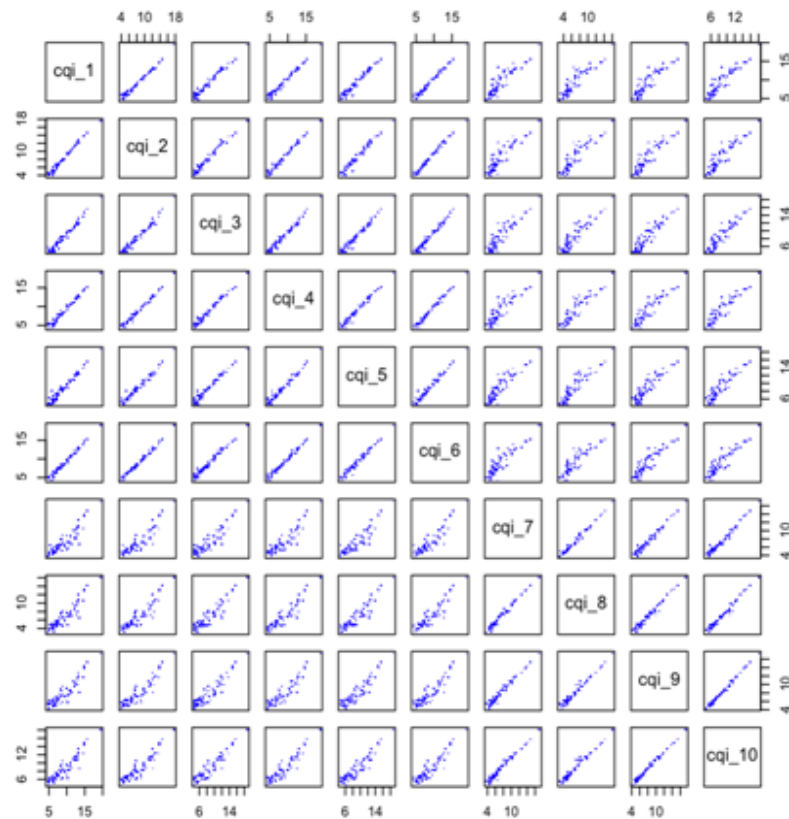

Homogeneity of CQI inter G6-slides

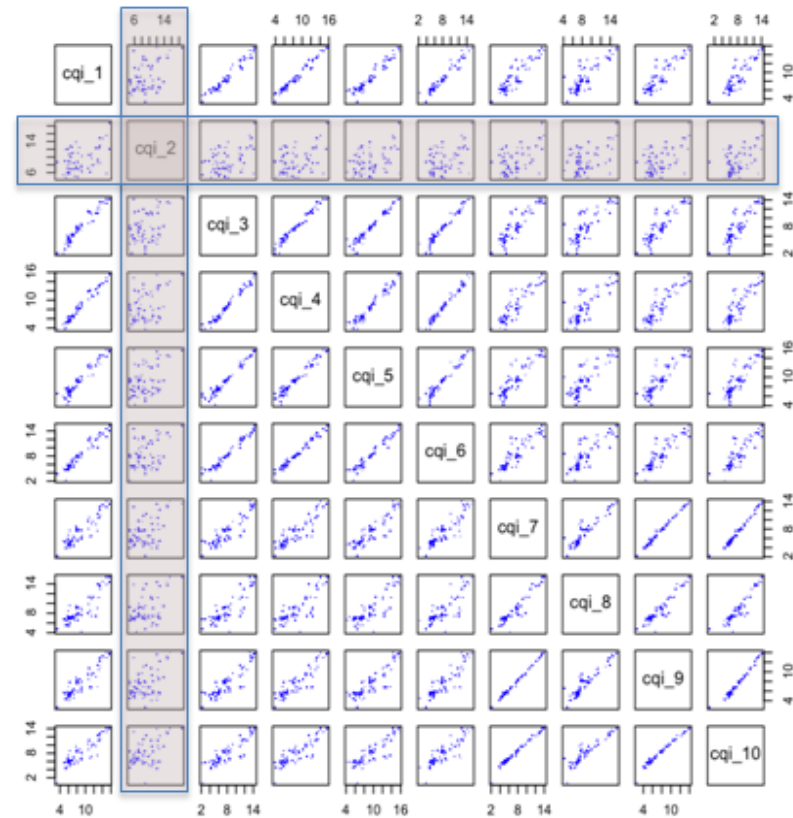

Supplement: Data S1 — CQI homogeneity between arrays. CQI was hybridized on each array (G6 and G7) in the very same conditions than the biological samples studied. Non-homogenous slide (G6, gray-highlighted) was extracted before analysis of the normalized data generated. [file Data_Sheet_1.PDF]
